# The Role of *N*-Glycosylation in the Intracellular Trafficking and Functionality of Neuronal Growth Regulator 1

**DOI:** 10.3390/cells11071242

**Published:** 2022-04-06

**Authors:** Gyuri Sim, Moonkyung Jeong, Hyunseok Seo, Jangrae Kim, Soojin Lee

**Affiliations:** Department of Microbiology and Molecular Biology, Chungnam National University, Daejeon 34134, Korea; tangerine_01@naver.com (G.S.); jmk3711@naver.com (M.J.); tjgustjr123@naver.com (H.S.); kjjanglae@naver.com (J.K.)

**Keywords:** *N*-glycosylation, neural cell adhesion molecule, membrane targeting, mutagenesis, ER

## Abstract

Neuronal growth regulator 1 (NEGR1) is a brain-enriched membrane protein that is involved in neural cell communication and synapse formation. Accumulating evidence indicates that NEGR1 is a generic risk factor for various psychiatric diseases including autism and depression. Endoglycosidase digestion of single NEGR1 mutants revealed that the wild type NEGR1 has six putative *N*-glycosylation sites partly organized in a Golgi-dependent manner. To understand the role of each putative *N*-glycan residue, we generated a series of multi-site mutants (2MT–6MT) with additive mutations. Cell surface staining and biotinylation revealed that NEGR1 mutants 1MT to 4MT were localized on the cell surface at different levels, whereas 5MT and 6MT were retained in the endoplasmic reticulum to form highly stable multimer complexes. This indicated 5MT and 6MT are less likely to fold correctly. Furthermore, the removal of two *N*-terminal sites N75 and N155 was sufficient to completely abrogate membrane targeting. An in vivo binding assay using the soluble NEGR1 protein demonstrated that glycans N286, N294 and N307 on the C-terminal immunoglobulin-like domain play important roles in homophilic interactions. Taken together, these results suggest that the *N*-glycan moieties of NEGR1 are closely involved in the folding, trafficking, and homodimer formation of NEGR1 protein in a site-specific manner.

## 1. Introduction

*N*-Linked protein glycosylation (*N*-glycosylation) occurs in all three life-form domains: eukaryotes, bacteria, and archaea [1]. It is closely implicated in the processing and functioning of secreted or cell surface proteins, including protein folding and targeting, and cell–cell interactions. Therefore, *N*-glycosylation defects are associated with a broad range of human diseases, including immune-related conditions and cancer [2]. The repertoire of *N*-glycans can be diverse depending on the tissue and cellular level, and it is closely linked to cell physiology and metabolism [3].

More than 100 congenital disorders of *N*-glycosylation are known, most of which (>80%) are related to severe impairment of the nervous system [4]. Genetic defects in the *N*-glycosylation pathway are usually associated with severe neurological abnormalities including structural abnormalities, myopathies, strokes, and epileptic seizures [5,6]. Glycan-containing molecules in the nervous system are highly involved in the development, regeneration, and synaptic plasticity of neurons by participating in cell–cell recognition and cell-matrix interactions [5]. It is known that a unique modification with polysialic acid plays a vital role in neural cell adhesion molecule-mediated neuronal development and plasticity [7].

Neuronal growth regulator 1 (NEGR1) is a brain-enriched glycosylphosphatidylinositol (GPI)-anchored membrane protein that was originally identified as a member of the IgLON superfamily containing limbic system-associated membrane protein (LAMP), opioid-binding cell adhesion molecule (OBCAM), and neurotrimin [8]. As a cell adhesion molecule, NEGR1 functions in human neuronal development and synaptic plasticity [9]. Recently, multiple genome-wide analyses have shown that genetic alterations in NEGR1 are associated with many major neurological disorders such as dyslexia [10], schizophrenia [11], Alzheimer’s disease [12], and depression [13]. Furthermore, we recently reported that Negr1-deficient mice displayed depressive-like behaviors [14] and increased fat mass [15].

In the present study, we characterized the glycan moieties of NEGR1 protein and discovered the role of each *N*-glycosyl residue in its folding, trafficking, and function. These results might contribute to disclose unidentified cellular functions and the regulatory pathways of NEGR1.

## 2. Materials and Methods

### 2.1. Cell Culture and Transfection

The HEK293, SHSY-5Y, and HeLa cells [16,17] used in this study were maintained in Dulbecco’s modified Eagle’s medium (Welgene, Gyeongsan, Korea) supplemented with 10% fetal bovine serum (Atlas Biologicals, Fort Collins, CO, USA) at 37 °C in a humidified atmosphere with 5% CO_2_, whereas SKOV3 cells [17] were maintained in RPMI-1640 medium (Welgene). Cells were transfected with either polyethylenimine (Sigma-Aldrich, St. Louis, MO, USA) or Lipofectamin 3000 (Invitrogen, Carlsbad, CA, USA) according to the manufacturers’ protocols. Male C57BL/6 mice were maintained on 12 h light/dark cycles in a controlled environment as previously described [15].

### 2.2. Plasmids and Site-Directed Mutagenesis 

To generate the pcDNA4-FLAG-NEGR1 construct, NEGR1 cDNA was subcloned into the pcDNA4/TO vector (Invitrogen) using *Afl*II and *Xba*I. Then, overlapping PCR reactions were conducted to insert the 3× FLAG sequence of the pcDNA3-3FLAG vector [17] into the C-terminus of the signal sequence (positions 1–39) of the NEGR1 gene. The protein disulfide isomerase A4 (*PDIA4*) gene was obtained from the 21C Human Gene Bank (KRIBB, Daejeon, Korea) and subcloned into the pKH3 vector [15] using *Hind*III and *Kpn*I. The NEGR1 mutants were generated using a PCR-based mutagenesis method and the pcDNA4-FLAG-NEGR1 plasmid.

### 2.3. Immunoblotting and Antibodies

Western blotting was performed as previously described [15]. Samples were lysed in NP-40 lysis buffer (50 mM Tris-HCl pH 8.0, 150 mM NaCl, 1% NP-40, 1 mM phenylmethylsulfonyl fluoride, and 5 mM EDTA) supplemented with a protease inhibitor cocktail (Sigma-Aldrich). For non-reducing gel electrophoresis, samples were not treated without *β*-mercaptoethanol. The following antibodies were used: FLAG (mouse monoclonal or rabbit monoclonal) from Sigma-Aldrich; NEGR1, HA, and *β*-actin from Santa Cruz Biotechnology Inc. (Dallas, TX, USA); Cy3 anti-mouse and Alexa Fluor 594 anti-human IgG from Jackson ImmunoResearch Laboratories (West Grove, PA, USA); and Fluorescein isothiocyanate (FITC) anti-mouse IgG from Invitrogen.

### 2.4. Homophilic Binding and Immunofluorescence Microscopy

For the homophilic binding assay, NEGR1-human Fc (hFc) construct was generated to produce hFc-conjugated soluble NEGR1 protein containing *N*-terminal three immunoglobulin (Ig)-like domains (1–314) [17,18]. After transfection into 293T cells, the culture medium was collected after 2 days. Then, NEGR1-hFc protein was purified using protein A sepharose (GE healthcare, Marlborough, MA, USA). After SKOV3 cells were transfected with NEGR1 wild type (WT) or NEGR1 mutants for 24 h and then incubated with a serum-free medium containing 30 μg/mL purified hFc or NEGR1-hFc [18] to for 1 h at 4 °C. After three washes, Alexa Fluor 594 anti-human IgG antibody (1:50) was added and incubated for 1 h at 4 °C. If necessary, cells were incubated with 4′,6-diamidino-2-phenylindole (DAPI, Sigma-Aldrich) and mounted with Vectashield mounting medium (Vector Laboratories, Burlingame, CA, USA).

For immunostaining, cells grown on coverslips were fixed in 4% paraformaldehyde in PBS for 15 min and permeabilized in 0.1% Triton X-100 for 10 min. After blocking cells in 10% CAS-Block (Invitrogen) in PBS for 30 min, they were incubated with the primary antibodies, followed by appropriate secondary antibodies. Imaging was performed using an Olympus BX51 fluorescence microscope (Olympus Co., Tokyo, Japan) or confocal laser scanning microscope (Zeiss LSM 880; Carl Zeiss, Oberkochen, Germany).

### 2.5. Protein Deglycosylation 

For enzymatic deglycosylation, peptide *N*-glycosidase F (PNGase F), *N*-neuraminidase, *O*-glycosylase, and endoglycosidase H (Endo H) were purchased from New England Biolabs (NEB, Frankfurt, Germany). Prior to enzyme reactions, samples were incubated in glycoprotein denaturing buffer (NEB) at 100 °C for 10 min. Enzyme digestion were carried out at 37 °C for 90 min, following the manufacturers’ instructions.

### 2.6. Cell Surface Biotinylation

Protein biotinylation was performed using the Pierce Cell Surface Protein Biotinylation and Isolation Kit (Thermo Fisher Scientific, Waltham, MA, USA) following the manufacturer’s protocol. Cells labeled with sulfo-NHS-SS-biotin were subjected to pulldown using streptavidin-agarose beads for 3 h at 4 °C. The beads were re-suspended in 5× sample loading buffer and heated at 100 °C for 10 min prior to SDS-PAGE.

### 2.7. Lipid Raft Fractionation and Cell Aggregation Assay

Lipid raft fractionation was performed using OptiPrep (Sigma-Aldrich) [16]. After lysates were adjusted to 40% OptiPrep, the samples were loaded into a centrifuge tube, and serially overlaid with 28% and 20% OptiPrep in PBS. After centrifugation for 3 h at 160,000× *g* in a SW 55 Ti rotor (Beckman Coulter, Brea, CA, USA), 300-μL fractions were collected from the top. Horseradish peroxidase-conjugated cholera toxin B subunit (Sigma-Aldrich) was used to detect ganglioside GM1, a raft marker. 

For the hanging drop assay, SKOV3 cells (3 × 10^4^) in 30 μL culture media were placed as hanging drops on the lid of a culture dish and allowed to aggregate for 16 h. Cell aggregates were counted under a microscope using the ImageJ software (NIH, Bethesda, MD, USA). All experiments were performed using triplicate samples and were repeated three times.

## 3. Results

### 3.1. Glycosylation Status of NEGR1 Protein

**Figure 1:**
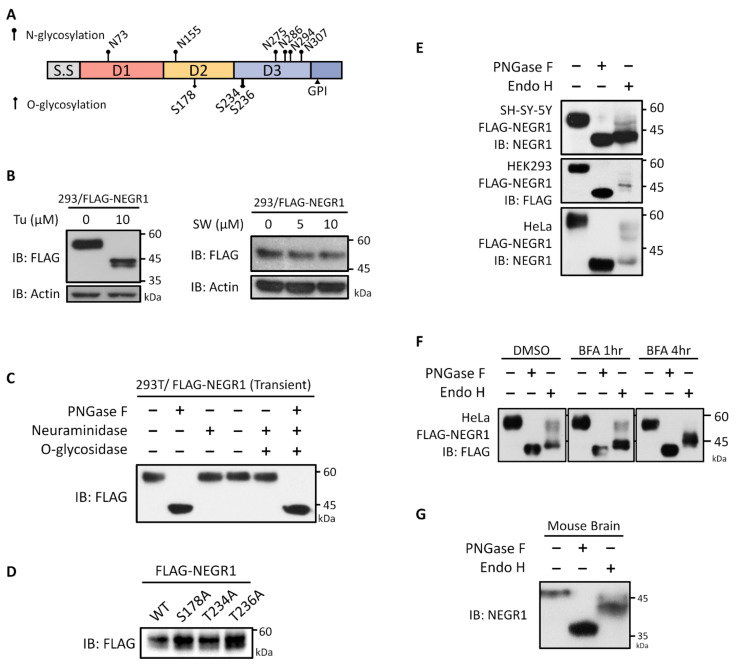
Characterization of glycosyl moieties of neuronal growth regulator 1 (NEGR1). (**A**) Schematic diagram of putative *N*- and *O*-glycosylation sites in NEGR1. S.S., Signal sequence; D1-3, Ig-like domains; GPI, glycosylphosphatidylinositol-anchoring site. (**B**) After transfection of FLAG-NEGR1, 293 cells were further incubated with different concentration of tunicamycin (Tu) or swainsonine (SW) for 2 h. (**C**) After transfection of FLAG-NEGR1 plasmids, 293T cell lysates were incubated with peptide *N*-glycosidase F (PNGase F), *N*-neuraminidase, or *O*-glycosidase for 90 min. (**D**) Western blotting of NEGR1 proteins that mutated at putative *O*-glycosylation sites. (**E**) At 24 h post-transfection of FLAG-NEGR1, cell lysates were obtained and incubated with PNGase F or Endo H for 90 min at 37 °C. (**F**) After transfection with FLAG-NEGR1 for 24 h, HeLa cells were further incubated with 5 µM brefeldin A (BFA) prior to enzyme digestion. (**G**) Enzymatic deglycosylation using mouse brain tissues obtained from 11-week-old male C57BL/6 mice.

To investigate the glycosyl moieties of NEGR1, putative *N*- and *O*-glycosylation sites were identified using the NetGGlyc 4.0 web-based prediction tool (http://www.cbs.dtu.dk/services/NetOGlyc/, accessed on 6 January 2020). Six putative *N*-glycosylation sites (N73, N155, N275, N286, N294, and N307) were identified (Figure 1A) and matched with other analysis tools (https://www.proteinatlas.org/, accessed on 6 January 2020). In contrast, the prediction scores of the putative *O*-glycosylation sites were below the level considered significant.

To examine the glycosylation status of NEGR1, HEK293 cells were transfected with FLAG-NEGR1 plasmid encoding NEGR1 fused with 3× FLAG at its *N*-terminus. Cells were then incubated with the *N*-glycan biosynthesis inhibitors, tunicamycin and swainsonine. Tunicamycin inhibits the formation of lipid-linked oligosaccharide precursors, whereas swainsonine blocks the processing of high-mannose to complex-type *N*-glycans. When cells were incubated with tunicamycin, the molecular weight of FLAG-NEGR1 (~58 kDa) decreased to ~43 kDa (Figure 1B), indicating that the total *N*-glycan moiety was ~15 kDa. When cells were treated with swainsonine, no differences in the size of NEGR1 were found up, to a concentration of 10 μM (Figure 1B), implying a very low content of complex-type oligosaccharides.

Next, we transfected FLAG-NEGR1 plasmids into 293T cells and subjected the cell lysates to deglycosylation. When incubated with PNGase F, which removes *N*-glycan moieties, the unglycosylated NEGR1 protein was ~43 kDa (Figure 1C), therefore displaying the same size as that observed in tunicamycin-treated cells. However, neither neuraminidase nor the combined neuraminidase plus *O*-glycosylase treatment changed the size of NEGR1, indicating it may not be *O*-glycosylated. To confirm this, we selected the three top-scoring putative *O*-glycosylation sites and changed them to alanine (Ala; S178A, T234A, and T236A). When expressed in 293T cells, no significant changes were observed in the size of the mutant proteins (Figure 1D), further demonstrating that NEGR1 is unlikely to contain *O*-glycosyl moieties.

Given that mature *N*-glycans are typically resistant to Endo H, digestion with this enzyme was performed after transient transfection of NEGR1-FLAG into three different cell lines (SHSY-5Y, HEK293, and HeLa). In contrast to the PNGase F digestion, which produced a single deglycosylated band, Endo H digestion produced multiple bands in a cell type-specific manner (Figure 1E), suggesting that NEGR1 contains both Endo H-sensitive and Endo H-resistant moieties. When cells were incubated with brefeldin A (BFA), an inhibitor of endoplasmic reticulum (ER)-to-Golgi transport, prior to Endo H treatment, the high molecular-weight products gradually disappeared (Figure 1F), implying that Endo H-resistant *N*-glycan moieties are produced in the Golgi apparatus. 

Finally, to examine endogenous proteins, endoglycosylase digestion was performed using whole brain tissue lysates obtained from 11-week-old male C57BL/6 mice (Figure 1G). Immunoblotting with anti-NEGR1 antibody showed that the mouse Negr1 band (~48 kDa) decreased to ~36 kDa upon PNGase F treatment. In contrast, several middle-sized bands were predominantly produced by Endo H digestion, implying that mature Negr1 protein has Endo H-sensitive moieties

### 3.2. Analysis of NEGR1 N-Glycosylation Mutants

**Figure 2:**
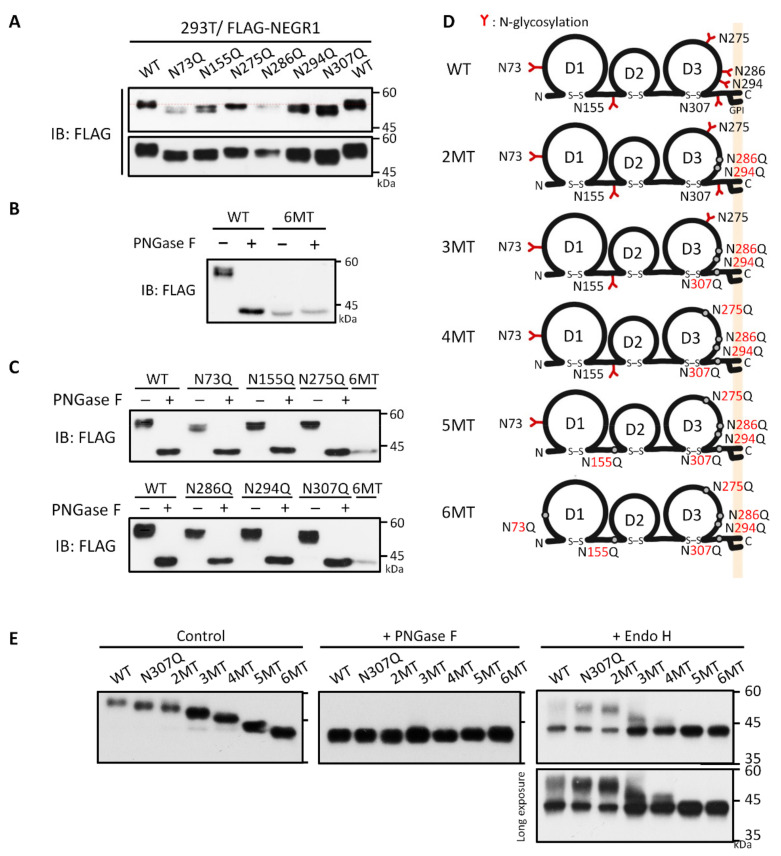
Generation of NEGR1 *N*-glycosylation mutants. (**A**) Immunoblotting of 293T cell lysates expressing *N*-glycosylation site-specific NEGR1 single mutants. (**B**) Deglycosylation of the NEGR1 6MT mutant, which contained all six *N*-glycosylation site mutations. The 293T cell lysates expressing wild type (WT) or 6MT NEGR1 were subjected to PNGase F digestion. (**C**) PNGase F digestion of each single *N*-glycosylation mutants. (**D**) Schematic representation of NEGR1 mutants (2MT–6MT), containing additive mutations in the following order: 286/294, 307, 275, 155, and 75. (**E**) NEGR1 WT and cumulative mutants were transfected into 293T cells and then subjected to enzymatic deglycosylation.

To investigate the role of each *N*-glycan on the NEGR1 protein, we produced six single *N*-glycosylation site mutants containing asparagine (Asn) to glutamine (Gln) mutations using the pcDNA4-3FLAG-NEGR1 plasmid. When expressed in 293T cells, all six mutants showed a decrease in the size of protein bands at different levels (Figure 2A). To determine whether NEGR1 might have extra *N*-glycosylation sites, we generated a mutant protein that contained mutations at all six predicted *N*-glycosylation sites (6MT). When expressed in 293T cells, the size of 6MT was exactly that of the unglycosylated WT NEGR1 (~43 kDa), and it did not change upon PNGase F digestion (Figure 2B). Furthermore, the individual single mutants generated the same band size as 6MT after PNGase F treatment (Figure 2C), demonstrating that NEGR1 has six *N*-glycans as predicted.

In addition to single mutants, we constructed a series of cumulative Asn mutants. *N*-glycosylation is concentrated at the C-terminus of all three consecutive Ig-like domains of NEGR1 (D1, D2, and D3, Figure 1A); D1 and D2 contained only one site each (N73 and N155, respectively), whereas four sites (N275, N286, N294, and N307) were located in D3. Based on our previous conclusion that the D3 domain is important for protein–protein interactions [14,16,18], we generated cumulative mutants starting from the residues in D3. A series of glycosylation mutant proteins (2MT–6MT) were generated with additive Asn to Gln mutations in the order 286/294, 307, 275, 155, and 73 (Table 1 and Figure 2D).

When mutants 2MT to 6MT were transiently expressed in 293T cells, we observed a stepwise decrease in size according to the number of mutations (Figure 2E). In addition, deglycosylated forms of the same size were generated from all these cumulative mutants. However, upon Endo H treatment, Endo H-resistant bands were generated only in mutants 2MT, 3MT, and 4MT. 5MT and 6MT showed a clear deglycosylated form, suggesting they may not be transported to the Golgi apparatus.

### 3.3. Membrane Localization of the NEGR1 Mutant Proteins 

To examine whether the *N*-glycosyl residues of NEGR1 could influence membrane trafficking, we performed immunofluorescence microscopy analysis using an anti-FLAG antibody. All single mutants were successfully visualized when expressed in HeLa cells (data not shown), demonstrating that the removal of a single *N*-glycosyl residue did not influence the membrane targeting of NEGR1.

**Figure 3:**
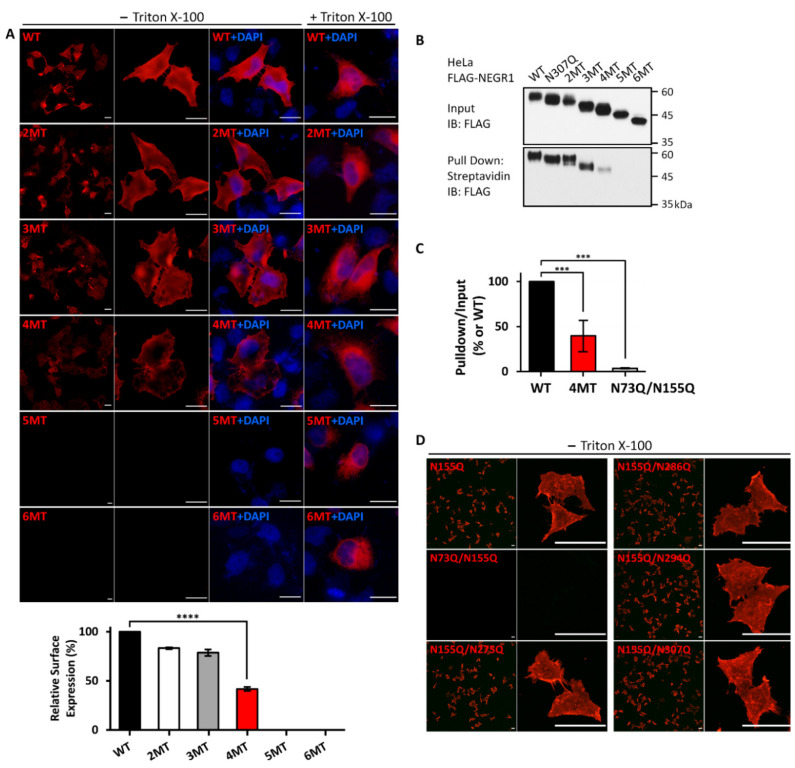
Membrane targeting of NEGR1 *N*-glycosylation mutants. (**A**) Immunofluorescence microscopy analysis of NEGR1 mutants. After transfection, HeLa cells were immunostained with anti-FLAG antibody with or without 0.1% Triton X-100. After incubation with Cy3-conjugated secondary antibody, images were captured using the Olympus BX51 fluorescence microscope. Scale bar = 50 μm. Cell surface expression was determined by calculating the relative fluorescence intensities in non-permeabilized cells after normalization to those in permeabilized cells. **** *p* < 0.0001. Error bars represent standard deviations (SD). (**B**) After transfection, HeLa cells were labeled with sulfo-NHS-SS-biotin using the Pierce Cell Surface Protein Isolation kit. Then, biotinylated proteins were isolated with streptavidin pulldown and subjected to immunoblotting using anti-FLAG antibody. (**C**) The surface to total ratio of 4MT and N73Q/N155Q mutant was calculated and normalized to that of WT. The data represent the average of three independent experiments ± SD. *** *p* < 0.001. (**D**) Membrane targeting of double mutants commonly containing N155Q. Cells were immunostained using anti-FLAG antibody under non-permeabilized conditions. Images were acquired using the Zeiss LSM 880 confocal microscope. Scale bar = 50 μm.

Next, we examined the membrane localization of the mutant proteins (2MT–6MT) in HeLa cells. The fluorescence signal levels of 2MT and 3MT were similar to those of WT NEGR1 (Figure 3A), whereas the fluorescence intensity of 4MT decreased to ~42% of that of WT. In contrast, almost no fluorescence signal was observed in 5MT- or 6MT-expressing HeLa cells. To confirm that 5MT and 6MT were expressed properly, we performed immunofluorescence microscopy analysis in the presence of 0.1% Triton X-100. Both 5MT and 6MT were observed under cell permeabilization (lower two panels, Figure 3A). The same results were observed in SKOV3 stable cells expressing WT or 6MT NEGR1 (Figure S1), indicating that 5MT and 6MT could lead to membrane targeting defects. 

To confirm the previous findings, we performed cell surface biotinylation using the membrane-impermeable sulfo-NHS-biotin after transfection of NEGR1 mutants into HeLa cells. Biotin-labeled proteins were collected using streptavidin-conjugated beads and visualized by immunoblotting with anti-FLAG antibody. The ratio of biotin-labeled 1MT (N307Q) and 2MT appeared similar to that of WT (Figure 3B), while 3MT showed a slightly reduced biotinylation efficiency. Moreover, the proportion of biotinylated 4MT was clearly reduced to ~38% of that of WT (Figure 3C). No biotin-labeled forms were observed for 5MT and 6MT, indicating that the cell membrane did not display these proteins.

Based on the dramatic difference in membrane targeting between 4MT and 5MT, we hypothesized that the newly mutated residue (N155) in 5MT could be critical for NEGR1 localization (Table 1). As the N155Q single mutant had no defect in membrane localization, we additionally generated double mutants that commonly contained N155Q and observed them under non-permeabilized conditions. Interestingly, only the N73Q/N155Q double mutant showed a clear defect in membrane localization, whereas the other double mutants were successfully targeted in the cell membrane (Figure 3D). In addition, no biotinylated protein was observed for N73Q/N155Q mutant (Figure 3C and Figure S1B). These results suggested that the glycosyl moieties at N73 and N155 may play key roles in NEGR1 trafficking.

### 3.4. Subcellular Localization of NEGR1 Mutants 

Given that the ER is a major organelle for regulating protein biosynthesis and folding, as well as the trafficking of membrane proteins [19], we examined whether the NEGR1 *N*-glycosylation mutant proteins were localized to the ER. To locate the ER, we generated pKH3-HA-PDIA4 plasmids encoding PDIA4, an ER marker. Next, the NEGR1 *N*-glycosylation mutant constructs 3MT to 6MT were co-transfected into SKOV3 cells together with HA-PDIA4 plasmids, and cells were subjected to double immunostaining with anti-FLAG and anti-HA antibodies.

**Figure 4:**
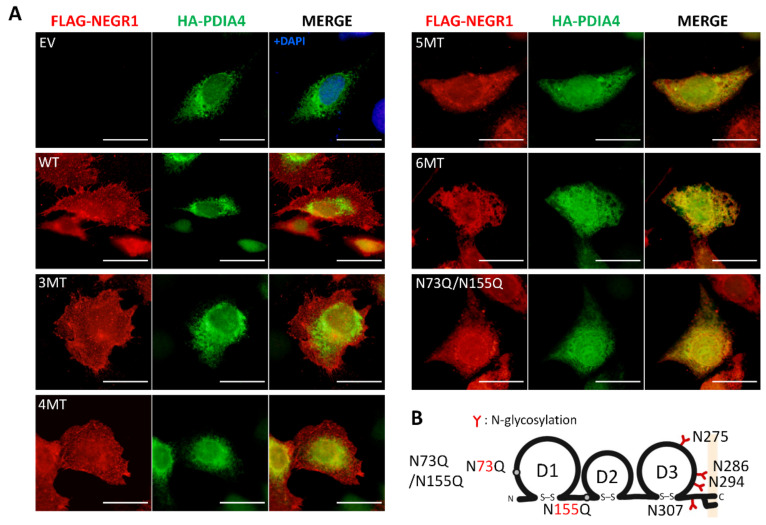
Subcellular localization of NEGR1 *N*-glycosylation mutants. (**A**) HeLa cells were co-transfected with FLAG-NEGR1 mutant constructs and HA-PDIA4 for 24 h. Then, cells were double-immunostained with anti-FLAG and anti-HA antibodies, followed by Cy3 anti-rabbit and fluorescein isothiocyanate anti-mouse secondary antibodies. Images were captured using the Olympus BX51 fluorescence microscope. Scale bar = 50μm. (**B**) Schematic representation of the N73Q/N155Q mutant.

NEGR1 WT, 3MT, and 4MT were observed throughout the cells and only partly co-localized with PDIA4 (Figure 4A). In contrast, the 5MT and 6MT signals almost completely overlapped with those of PDIA4. Furthermore, the previously identified trafficking-defective double mutant N73Q/N155Q (Figure 4B) co-localized with PDIA4. These data indicate that 5MT, 6MT, and N73Q/N155Q are retained in the ER and are defective in ER-to-Golgi trafficking.

### 3.5. Characterization of NEGR1 N-Glycosylation Mutants 

**Figure 5:**
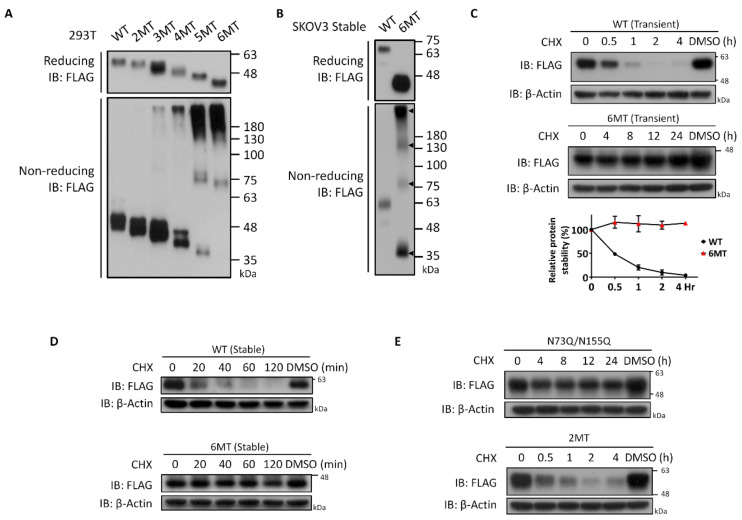
Multimer formation and protein stability of NEGR1 mutants. (**A**) Non-reducing SDS-PAGE analysis. The 293T cell lysates expressing NEGR1 mutant proteins were subjected to SDS-PAGE under non-reducing conditions, followed by immunoblotting using anti-FLAG antibody. (**B**) Non-reducing SDS-PAGE using SKOV3 stable cells expressing WT or 6MT. (**C**) To determine the half-lives of NEGR1 WT and 6MT, 293T cells were incubated in the presence of cycloheximide (CHX, 50 μg/mL) after transfection. Intensities of bands were quantified by ImageJ and normalized to *β*-actin. Data are means ± standard deviation (*n* = 3). (**D**) Half-life determination using SKOV3 cells stably expressing NEGR1 WT or 6MT. (**E**) After 293T cells were transfected with N73Q/N155Q and 2MT for 24 h, cells were incubated with 50 μg/mL CHX.

To characterize the NEGR1 *N*-glycosylation mutant proteins, we analyzed the multimeric status of each mutant protein. After transfection into 293T cells, cell lysates were subjected to SDS-PAGE under non-reducing conditions. Unexpectedly, high molecular weight complex forms started to appear in 3MT (Figure 5A). Only a minor portion of the total protein was considered complex for 3MT and 4MT, while the majority of 5MT and 6MT proteins were found in the complex form. In addition, when performing the same experiment with SKOV3 cells stably expressing 6MT, we found multimer bands corresponding to monomer, dimer, tetramer, and multimer complexes (Figure 5B). These results suggested that ER-retained 5MT and 6MT might be cross-linked by intermolecular disulfide bonds, possibly due to incorrect protein folding.

Next, we examined the protein stability of the *N*-glycosylation-defective NEGR1 mutants. After transfection of NEGR1 WT and 6MT into 293T cells, these were incubated with the protein synthesis inhibitor cycloheximide. The NEGR1 WT protein rapidly disappeared upon cycloheximide treatment, with a half-life of ~30 min (Figure 5C). However, the half-life of ER-resident 6MT significantly increased and it remained intact up to 24 h after cycloheximide addition. Similar results were obtained when the half-life was measured in SKOV3 stable cells, showing that 6MT was highly stable compared to WT (Figure 5D). Similar to WT protein, 2MT rapidly degraded, whereas the trafficking-defective N73Q/155Q double mutant showed a prolonged half-life (Figure 5E). Taken together, these results indicate that ER-retained NEGR1 mutant proteins form a complex using disulfide bonds to dramatically increase their long-term stability.

### 3.6. Homophilic Interaction of NEGR1 N-Glycosylation Mutants 

Given that NEGR1 functions as a cell adhesion molecule that is involved in cell–cell homophilic and heterophilic interactions [20], we evaluated whether glycosylation status may affect homodimer formation on the cell surface. After transfection of NEGR1 constructs into SKOV3 cells, these were incubated with a serum-free medium containing purified NEGR1-hFc. The bound NEGR1-hFc was visualized with anti-human IgG antibody under non-permeabilized conditions.

**Figure 6:**
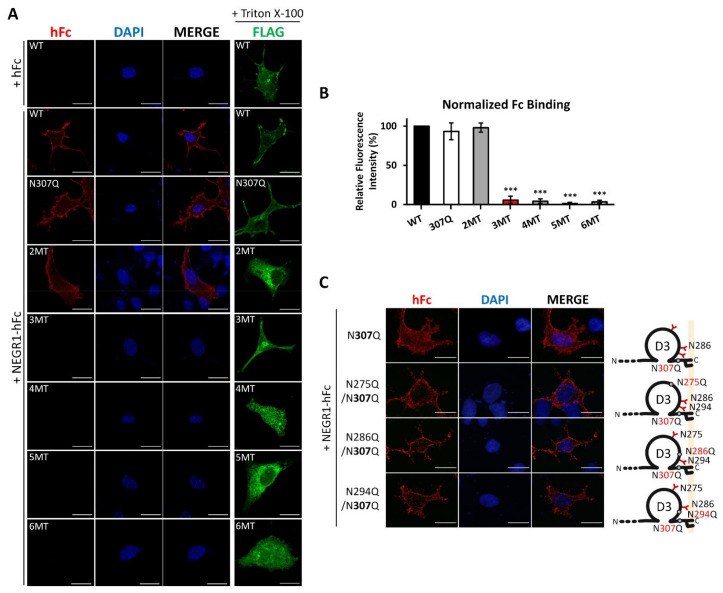
Homophilic binding activities of NEGR1 mutants. (**A**) In situ homophilic interaction using soluble NEGR1 protein. After SKOV3 cells were transfected with NEGR1 WT and mutants for 24 h, cells were incubated in a serum-free medium containing purified hFc or NEGR1-hFc (30 μg/mL) for 1 h at 4 °C. After fixation, bound NEGR1-hFc was visualized by incubation with Alexa Fluor 595 anti-hFc antibody (1:50) for 1 h. To verify the protein expression, cells were separately immunostained with anti-FLAG antibody under cell permeabilization conditions. Images were obtained using the Zeiss LSM 880 confocal microscope. (**B**) Homophilic interaction was determined by calculating the relative fluorescence intensities of cells stained with anti-hFc antibody after normalization to those with anti-FLAG antibody. Data are means ± standard deviation *** *p* < 0.001 (**C**) Double mutants commonly containing N307Q were generated, and an in-situ binding assay was performed using NEGR1-hFc.

We detected fluorescent signals in NEGR1 WT-expressing cells when these were treated with NEGR1-hFc, but not when they were treated with the hFc control (Figure 6A). A similar hFc signal was observed in 2MT-expressing cells. However, fluorescence signals were barely detected in 3MT. Considering that 3MT showed no defects in membrane targeting (Figure 3A,B), it was quite unexpected that 3MT showed defects in homophilic interactions. The protein expression of each mutant was confirmed by performing immunofluorescence microscopy analysis in the presence of 0.1% Triton X-100 (fourth column, Figure 6A).

As 3MT contains an additional N307Q mutation relative to 2MT, we tested whether N307 is critical for homodimer formation. Based on the successful binding of NEGR1-hFc to the N307Q mutant (Figure 6B), we generated double mutants containing one mutation in D3 (N275Q, N286Q, or N294Q) in addition to N307Q. Immunofluorescence microscopy showed that all these double mutants successfully interacted with NEGR1-hFc (Figure 6B), implying that the loss of NEGR1 homophilic binding was induced by glycosylation defects in the three Asn residues (N286, N294, and N307).

### 3.7. Cell Aggregation Assay and Lipid Raft Fractionation 

**Figure 7:**
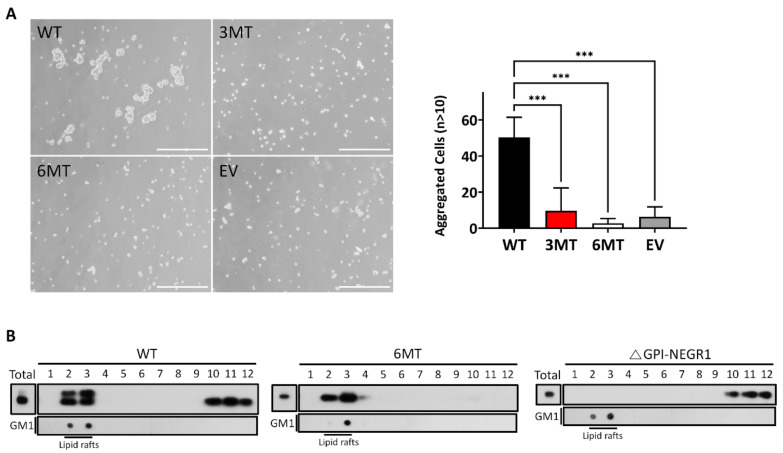
Hanging drop assay and lipid raft fractionation. (**A**) SKOV3 cell suspensions (3 × 10^4^ in 30 μL culture medium) were placed as hanging drops on the lid of a Petri dish and allowed to aggregate for 16 h. Cell aggregates containing more than ten cells were counted under the microscope using ImageJ software. Data are means ± standard deviation of three independent experiments. *** *p* < 0.001. Scale bar = 500 μm. (**B**) After 293T cells were transfected with WT, 6MT, or a truncated *Δ*GPI NEGR1, lipid raft fractionation was conducted using ultracentrifugation with OptiPrep. As a raft marker, ganglioside GM1 was also visualized using horseradish peroxidase-conjugated cholera toxin B.

To evaluate whether the changes in homophilic interactions could affect cell–cell adhesion, a hanging drop aggregation assay was performed. After SKOV3 cells were transfected with WT, 3MT, or 6MT, the cells were placed in hanging drops inside the culture dish lid. After 16 h, we observed cell clumps containing more than ten cells in the NEGR1 WT-transfected sample, whereas fewer aggregated cells were found in 3MT-transfected cells, similar to 6MT or vector-transfected controls. These data supported the lower cell–cell attachment in 3MT than in WT NEGR1 (Figure 7A).

NEGR1 is a GPI-anchored protein localized in membrane rafts. To examine if the glycosylation status might change raft association, we isolated the lipid raft fraction using 293T cells transfected with WT, 6MT, or a truncated *Δ*GPI-NEGR1 lacking the C-terminal GPI-anchoring region [17]. While none of the *Δ*GPI-NEGR1 proteins was observed in the raft fraction, WT NEGR1 was enriched in the membrane rafts (Figure 7B). In particular, 6MT mutant proteins were exclusively localized in lipid rafts, suggesting that 6MT has no defects in GPI-anchoring and the raft association.

## 4. Discussion

Approximately one-third of mammalian proteins are produced in the ER and delivered to the membrane or secretory vesicles from the Golgi apparatus [21]. Most membrane or secretory proteins are *N*-glycosylated and synthesized in the ER, and the immature glycans (mannose-rich) are trimmed down and modified in the Golgi apparatus during the secretory process [21]. Although these core-glycosylated *N*-glycans are rarely observed in typical mammalian cells, high levels of core-glycosylated *N*-glycans are present in the neuronal plasma membrane [22].

In cultured neurons, core-glycosylated proteins are displayed at levels similar to those of conventional mature (hybrid and complex type) *N*-glycans [22]. In particular, a substantial fraction of key neuronal surface proteins, including GABA and glutamate receptor subunits, are Endo H-sensitive core-glycosylated forms [23]. Due to the unique, highly extended structures of neuronal cells and their lack of canonical Golgi compartments in distal dendrites or axons, it is believed that the newly synthesized glycoproteins are trafficked via a so-called “Golgi bypass” pathway in this region [24].

In the present study, we revealed that human NEGR1 has six *N*-glycosylated moieties with diverse molecular masses and identified residues that are important for its stability, trafficking, and homophilic activities. Human NEGR1 proteins, as well as mouse brain Negr1, showed high levels of Endo H-sensitive glycans (Figure 1E,G). Based on the high expression of NEGR1 in the neuronal somata and dendritic synaptic vesicles [25], NEGR1 may be trafficked via Golgi-independent secretory pathways.

A previous site-specific glycosylation analysis using mass spectrometry revealed the glycosylation moieties of four IgLON family proteins: LAMP, OBCAM, neurotrimin, and the rat NEGR1 homologue, kilon (a kindred of IgLON) [26]. *N*-Glycosylation sites near the *N*-terminus in LAMP, OBCAM, and neurotrimin commonly contain disialic acids, which might play an important role in cell−cell interactions. However, the *N*-terminal glycosylation site (N36) of kilon was determined to be a high mannose-type glycan, suggesting that kilon may have a unique glycan structure. Moreover, the core-glycosylated moieties were more frequently observed in kilon than in the other IgLON members, which is consistent with our findings that NEGR1 has a substantial fraction of Endo H-sensitive glycans. 

Cell surface staining and biotinylation studies revealed that the removal of two glycosyl moieties near the *N*-terminus (N73 and N155) was sufficient to block their membrane targeting (Figure 3D). Although 3MT and 4MT (N275Q/N286Q/N294Q/N307Q), which contain mutations in the D3 domain of NEGR1, showed progressively attenuated membrane targeting, the fifth mutation (N155Q in the D2 domain) of 5MT completely abrogated membrane localization. These results were confirmed by non-reducing SDS-PAGE (Figure 5A). The percentage of high molecular weight multimeric forms dramatically increased from 5MT. Since none of the other double mutants showed the same defects as N73Q/N155Q (Figure 3D), we suggest that the glycans at the *N*-terminal D1 and D2 domains have more important functions than those in D3 for ER-to-Golgi trafficking of NEGR1. 

We examined the homophilic binding between membrane-associated NEGR1 mutant proteins and soluble NEGR1-hFc protein (Figure 6). We observed that 2MT (N286Q/N294Q) bound normally to soluble NEGR1, contrarily to that found for 3MT. The hanging drop assay also revealed that 3MT-transfected cells formed significantly fewer cell aggregates than did WT-transfected cells (Figure 7A). The 3MT had an additional mutation at N307, localized close to the GPI-anchor (Figure 1A). 

Previously, we reported several NEGR1-interacting proteins such as Niemann Pick C, type 2 (NPC2) [16], Na^+^/K^+^-ATPase beta subunit (ATP1B1) [18], and leukemia inhibitory factor receptor (LIFR) [14]. NPC2 is a soluble late endosomal protein, whereas ATP1B1 and LIFR are membrane-associated proteins. A recent structural analysis proposed that NEGR1 forms homodimers using D1 domains [27]. However, our previous domain mapping studies showed that, whether secreted or membrane-bound, these NEGR1-binding proteins exhibited a clear preference to the D3 domain of NEGR1, although they still showed minor binding affinities for D1 and D2. Because 3MT showed no significant defects in membrane localization (Figure 3A), we hypothesize that the glycosyl moieties in the membrane-proximal region may not play an important function in NEGR1 trafficking but play critical roles in protein–protein interactions.

ER-resident mutants, such as 5MT, 6MT, and N73Q/N155Q, showed dramatically increased protein stability. The half-life of NEGR1, either stably or transiently expressed, was estimated to be ~30 min (Figure 5C,D), while the level of 6MT did not decrease even 24 h after cycloheximide addition. Interestingly, 6MT proteins were successfully localized in lipid rafts (Figure 7B) and GPI anchoring was not affected by the absence of glycosyl moieties. However, the proper raft association of 6MT did not protect it from cross-linking to form a multimeric structure (Figure 5A). The formation of abnormal intermolecular disulfide bonds in the ER-retained mutants may indicate that these proteins did not fold correctly in the ER. The ER is known to have a unique quality control mechanism [28]. The *N*-glycans required for the ER-to-Golgi trafficking of NEGR1 may be tightly connected with the formation and/or maintenance of the proper conformation of NEGR1.

*N*-Glycosylation controls the function of many key players implicated in the regulation of synapse formation and plasticity [29]. While the majority of the studies on *N*-glycosylation have focused on the functions in protein folding and quality control inside the cells, much less is known about the functions of *N*-glycosylation outside of the cells [29]. Although further investigation is needed to determine the precise glycan structures and their specific functions, the *N*-glycosylation status of NEGR1 may be significantly related to its diverse functions in the nervous system. Overall, we propose that *N*-glycosylation of NEGR1 is closely involved in protein stability and intracellular trafficking, as well as in protein–protein interactions, which may contribute to better understanding of the roles and control mechanisms of NEGR1.

## 5. Conclusions

We aimed to explore the roles of *N*-glycosylation in the processing and functionality of NEGR1. By analyzing a series of NEGR1 *N*-glycosylation mutants, we suggested that the glycans near the *N*-terminus have important functions in NEGR1 stability and its ER-to-Golgi trafficking, while those near the C-terminus play critical roles in homophilic interaction. Overall, *N*-glycosylation of NEGR1 is closely involved in its folding, trafficking, and homodimer formation, which may be useful for understanding the diverse functions and as yet unidentified regulatory mechanisms of NEGR1.

## Figures and Tables

**Table 1:** *N*-Glycosylation mutants of NEGR1.

Mutant Name	Mutation Sites
Single mutant	N73Q, N155Q, N275Q, N286Q, N294Q, or N307Q
N73Q/N155Q	N73Q + N155Q
2MT	N286Q + N294Q
3MT	N286Q + N294Q + N307Q
4MT	N286Q + N294Q + N307Q + N275Q
5MT	N286Q + N294Q + N307Q + N275Q + N155Q
6MT	N286Q + N294Q + N307Q + N275Q + N155Q + N73Q

## Data Availability

The data presented in this study are available from the corresponding author upon reasonable request.

## Supplementary Materials

The following supporting information can be downloaded at: https://www.mdpi.com/article/10.3390/cells11071242/s1, Figure S1: Cell surface expression of NEGR1 mutants.
