# Supplementary figures and images for: The Role of N-Glycosylation in the Intracellular Trafficking and Functionality of Neuronal Growth Regulator 1
Source: Cells. 2022 Apr 6;11(7):1242. doi: 10.3390/cells11071242 (PMC8997467; doi:10.3390/cells11071242)

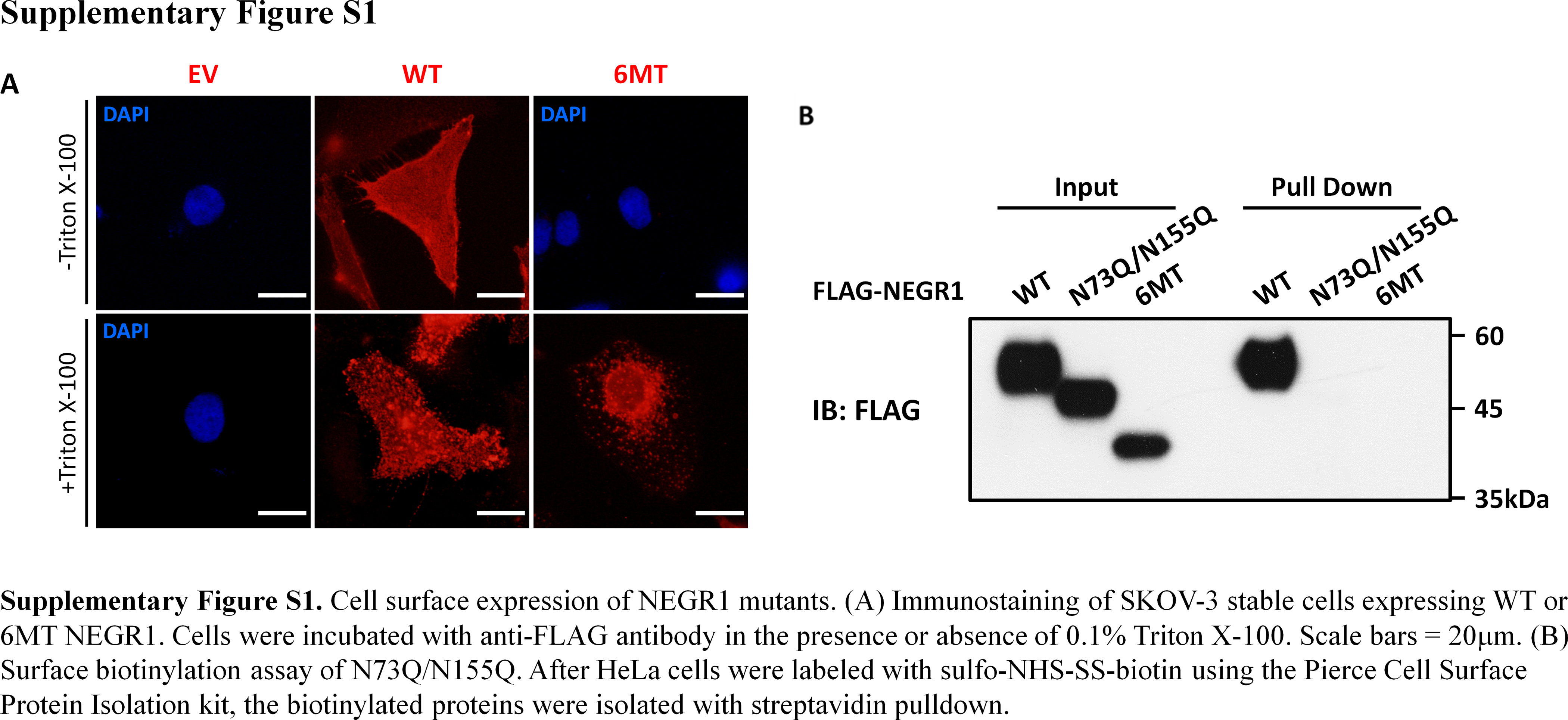

Supplement: Supplementary file 1 [file cells-11-01242-s001.zip › cells-1635131-Figure S1.tif]
